# The biological function and potential mechanism of long non‐coding RNAs in cardiovascular disease

**DOI:** 10.1111/jcmm.15968

**Published:** 2020-10-13

**Authors:** Chengmeng Zhang, Bing Han, Tongda Xu, Dongye Li

**Affiliations:** ^1^ Institute of Cardiovascular Disease Research Xuzhou Medical University Xuzhou China; ^2^ Department of Cardiology Xuzhou Central Hospital Xuzhou China; ^3^ Department of Cardiology The Affiliated Hospital of Xuzhou Medical University Xuzhou China

**Keywords:** cardiovascular diseases, long non‐coding RNA, microRNA, post‐transcriptional regulation, transcriptional regulation

## Abstract

Long non‐coding RNAs (lncRNAs), as part of the family of non‐protein‐coding transcripts, are implicated in the occurrence and progression of several cardiovascular diseases (CVDs). With recent advances in lncRNA research, these molecules are purported to regulate gene expression at multiple levels, thereby producing beneficial or detrimental biological effects during CVD pathogenesis. At the transcriptional level, lncRNAs affect gene expression by interacting with DNA and proteins, for example, components of chromatin‐modifying complexes, or transcription factors affecting chromatin status. These potential mechanisms suggest that lncRNAs guide proteins to specific gene loci (eg promoter regions), or forestall proteins to specific genomic sites via DNA binding. Additionally, some lncRNAs are required for correct chromatin conformation, which occurs via chromatin looping in enhancer‐like models. At the post‐transcriptional level, lncRNAs interact with RNA molecules, mainly microRNAs (miRNAs) and mRNAs, potentially regulating CVD pathophysiological processes. Moreover, lncRNAs appear to post‐transcriptionally modulate gene expression by participating in mRNA splicing, stability, degradation and translation. Thus, the purpose of this review is to provide a comprehensive summary of lncRNAs implicated in CVD biological processes, with an emphasis on potential mechanisms of action.

## 1 INTRODUCTION

The deep sequencing surveys of mammalian genomes have revealed pervasive and complex transcription processes. The majority of the human genome is transcriptionally active, but only a small fraction (<3%) is translated into protein, while the remainder is transcribed as non‐coding RNAs (ncRNAs).^1^ This discovery has led to an explosion in transcriptomics and has revealed that ncRNAs exert comprehensive effects on cellular biological processes. Conventionally, ncRNAs are characterized as RNAs with extremely limited protein‐coding potential. Different categories of ncRNAs exist and are based on nucleotide (nt) length; ncRNAs are roughly divided into small ncRNAs and long ncRNAs (lncRNAs). The former group (<60nt) exists as miRNAs and short interfering RNAs (siRNAs). Of these, miRNAs measure approximately ~22nt in length and regulate the gene expression of both protein‐coding and non‐coding genes, leading to post‐transcriptional silencing or infrequent activation.^2^, ^3^ It is acknowledged that miRNAs are involved in a variety of physiological and pathological processes. In contrast, lncRNA research is in its infancy. However, thanks to the development of RNA‐sequencing technologies and genome‐wide analyses, thousands of lncRNAs have been unveiled. Despite intense technological efforts, only a small proportion of identified lncRNAs have been functionally annotated. In the following, we review the generally accepted categorization and function of lncRNAs, and explore the potential regulatory mechanisms of cardiovascular‐related lncRNAs in cardiovascular physiology and pathology.

## 2 LncRNA CLASSIFICATION: LOCATION AND FUNCTION

LncRNAs represent a subgroup of ncRNAs ranging from 200nt to ~100 kilobases (Kb) in length. However, they lack open reading frames (ORFs).^4^ Until now, lncRNA classification criteria have not been unified. LncRNA nomenclature is primarily based on their empirical features, including origin of transcription, molecular function as well as cellular localization.^5^ Through greater understanding of RNA‐sequencing data/technologies, more than 50,000 lncRNAs, from intronic, exonic or intergenic regions, have been identified in many different human tissues.^6^ The majority of lncRNA genomic loci rely on intergenic regions; some are found in introns of coding genes,^7^ or they originate from enhancer gene regions (enhancer‐derived lncRNAs, elncRNAs). With the identification of circular RNAs (circRNAs), which are processed by the intron back‐splicing, it has been recognized that the genomic structure of lncRNAs is more than just linear.^8^

Similarly, lncRNAs are also categorized by their molecular function: (a) Signal lncRNAs can serve as signalling molecules in response to cellular cues or particular stimuli; they participate in tissue molecular signalling in a time‐dependent manner. (b) Decoy lncRNAs regulate transcription through competitively binding and sequestering RNA‐binding proteins (RBPs) or miRNAs targets. (c) Guide lncRNAs serve as molecular guides to recruit chromatin‐modifying enzymes to target gene loci. (d) Scaffold lncRNAs are linkers that assemble multiple proteins to form ribonucleoprotein complexes (RNPs). (e) Enhancer lncRNAs gather at promoter and enhancer regions via chromosomal loops, to enhance transcription.^9^

Typically, lncRNAs are transcribed by RNA polymerase II or III (RNA pol II/ III) molecules, which are matured by selective cleavage and subjected to 5' end capping and polyadenylation processing.^10^ Based on their cellular localization, lncRNAs can be divided into two major categories: nuclear and cytoplasmic. Nuclear‐localized lncRNA transcripts have structural and regulatory roles and affect chromatin status by interacting with chromatin‐modifying complexes, thereby controlling gene transcriptional activities.^11^, ^12^ Cytoplasmic lncRNAs complement with mRNAs to form double‐stranded RNA molecules, which interfere with mRNA stability and translation, protein localization and turnover and other signalling pathways.^13^ They also function as endogenous sponges for miRNAs, thereby preventing translational inhibition or mRNA degradation.^14^

## 3 LncRNAS AS EMERGING REGULATORS IN CARDIOVASCULAR BIOLOGY

Far removed from the concept of ‘junk RNA’, lncRNAs are believed to be functional regulators that modulate normal development and disease processes in the cardiovascular system. By monitoring lncRNA differential expression, studies have identified a number of lncRNAs that play crucial roles in regulating gene expression programmes during normal cardiovascular development and CVD‐related pathogenesis.^15^, ^16^ Globally, CVDs are a major cause of mortality and generate huge health and financial burdens worldwide.^17^ CVD pathogenesis arises in response to a variety of pathological stimuli such as apoptosis, necrosis, autophagy and cardiomyocyte hypertrophic mechanisms, and the abnormal proliferation and differentiation of cardiac fibroblasts, vascular smooth muscle cells (VSMCs) and endothelial cells (ECs). LncRNAs participate in the initiation and progression of diverse CVDs, by modulating a wide variety of cardiovascular processes, including myocardial infarction (MI), cardiac hypertrophy and fibrosis, atherosclerosis, arrhythmias and heart failure (HF).^18^, ^19^

## 4 MECHANISMS OF ACTION OF CARDIOVASCULAR‐RELATED LncRNAS

In recent years, a number of studies have shown that lncRNAs play pivotal mechanistic roles in regulating gene expression, through multiple mechanisms, at different levels. The underlying molecular mechanisms underpinning several lncRNAs have not yet been elucidated. However, the exploration of cardiovascular‐related lncRNAs is still in its early stages. Therefore, understanding mechanisms behind these functional transcripts is a major disease challenge. In the following sections, we summarize recent discoveries linking lncRNA biological functions to potential CVD mechanisms, at transcriptional and post‐transcriptional levels.

### 4.1 LncRNAs in transcriptional regulation

#### 4.1.1 LncRNAs transcriptional regulation via the recruitment of multiple chromatin modifiers

**FIGURE 1:**
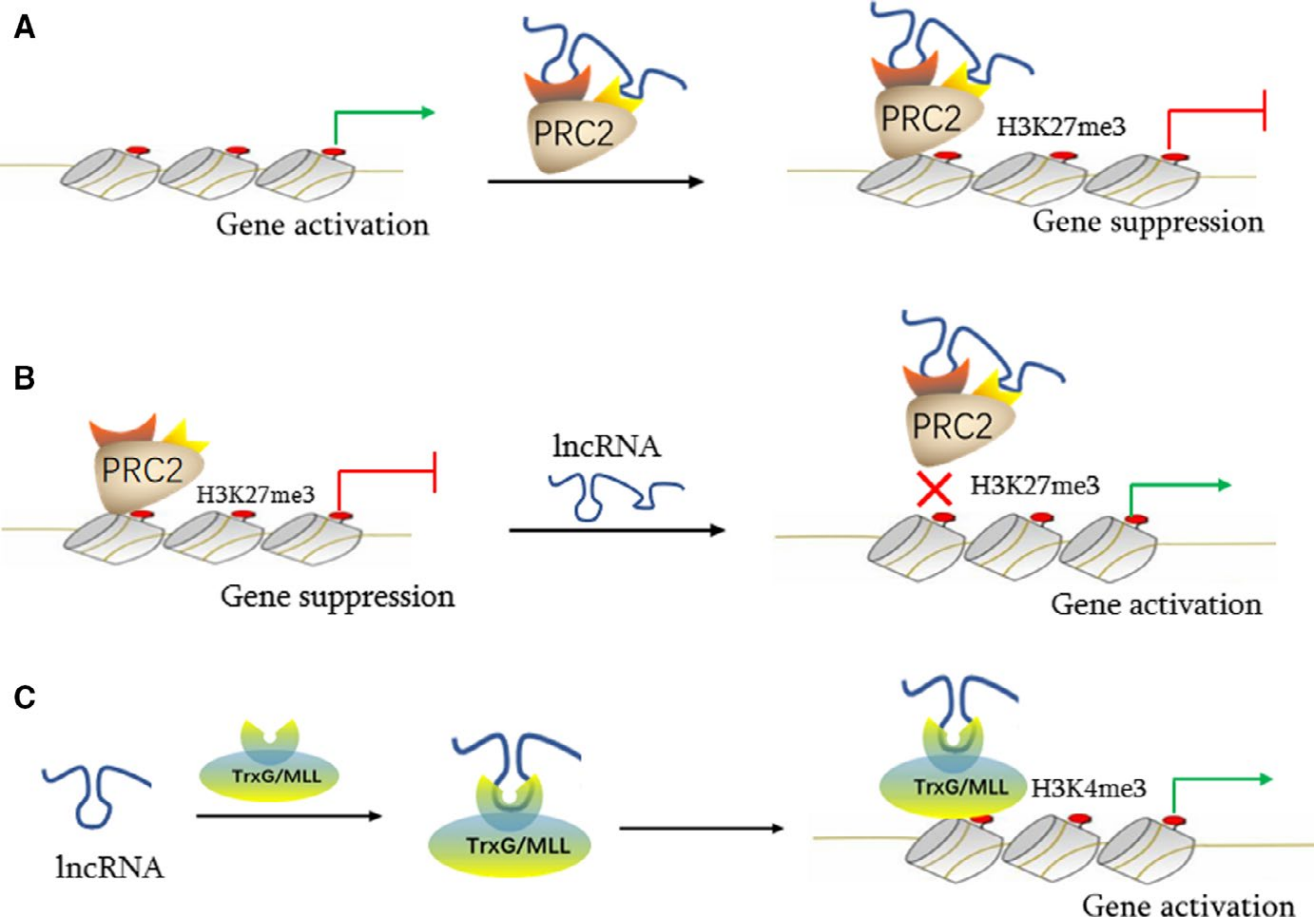
Transcriptional regulation of cardiovascular lncRNAs via interactions with multiple chromatin‐modifying complexes. The mode of action of cardiac lncRNAs, such as Fendrr, is shown in the guiding scaffold model (A, C); In the trapped scaffold model, Bvht and Chaer detach histone‐modifying enzymes, such as PRC2, from target genome sites (B). lncRNA, long non‐coding RNA; PRC, polycomb repressive complex; TrxG/MLL, trithorax/mixed lineage leukaemia

The regulation of chromatin structure is of great importance for gene transcription in eukaryotes. Enzymes that catalyse chromatin structural changes mainly include histone‐modified and chromatin‐remodelling complexes. In recent years, lncRNAs have been shown to regulate the access or dismissal of multiple chromatin‐modifying complexes from chromatin, and activating or inhibiting gene expression at the transcriptional level. Such regulatory modes are common for lncRNAs, as approximately 40% of these molecules interact directly with diverse chromatin‐modifying complexes.^20^ Mechanistically, lncRNAs function as scaffolds to assemble chromatin‐modifying enzymes into complexes, facilitating lncRNAs as guides to recruit these complexes to specific genomic loci, thereby altering chromatin expression. An alternative scenario could be that other lncRNAs interact with chromatin‐modifying complexes by acting as decoys to impede the binding of chromatin modifiers to target genomic loci, in turn affecting transcriptional processes (Figure 1).^21^

##### 4.1.1.1 Interactions with histone‐modifying complexes

Approximately 40% of lncRNAs are directly related to diverse histone‐modifying complexes, including PRC2, MLL, LSD1, WDR5 and others.^22^, ^23^ It is accepted that lncRNAs serve as scaffolds to coordinate the recruitment of protein complexes to target loci, to change the chromatin or DNA state.^21^ A group of cardiovascular‐related lncRNAs (ie Chaer, Bvht, Fendrr, etc) interplay with histone‐modified enzymes to affect gene transcription during normal cardiovascular development and disease processes. In 2016, Wang *et al* identified Chaer, a heart‐enriched lncRNA that appeared to contribute to cardiac hypertrophy and induce genes involved in hypertrophy. These authors detected a dynamic change in histone H3 lysine 27 methylation (H3K27me), following phenylephrine (PE) treatment in rat ventricular myocytes. A Chaer knockdown in cultured cardiomyocytes increased H3K27 trimethylation (inactivating marker), whereas Chaer overexpression significantly reduced trimethylation levels at the H3K27 site, suggesting a negative regulatory role for this lncRNA in H3K27 methylation in cardiomyocytes. Following this observation, Wang *et al* further verified that Chaer negatively regulated H3K27 methylation, by directly interacting with the EZH2 subunit of polycomb repressor complex 2 (PRC2), which is a specific histone methyltransferase (core subunits are EZHZ and EED) that catalyses histone H3 lysine 27 trimethylation. By acting as a molecular decoy, Chaer bound to PRC2 and impeded its targeting to loci, thereby inhibiting H3K27me at promoter regions of target genes implicated in cardiac hypertrophy.^24^ In other work, Chaer in patients with atherosclerosis was highly expressed when compared with healthy individual, suggesting that Chaer promoted atherosclerosis by mediating PRC2 activity via the mTOR signalling pathway.^25^ Additionally, Klattenhoff *et al* identified a mouse‐specific, heart‐related lncRNA termed Braveheart (Bvht), which was required for cardiovascular lineage commitment and cell differentiation. LncRNA‐Bvht appeared to interplay with PRC2 to regulate target gene transcriptional programmes.^26^

The lncRNA, Fendrr, also plays a critical regulatory role in embryonic cardiac differentiation.^27^, ^28^ LncRNA‐Fendrr non‐specifically interacted with either the PRC2 or WDR5 component of the histone methyltransferase complex, trithorax/mixed lineage leukaemia (TrxG/MLL). In contrast to the PRC2‐catalysed repressive histone modification, H3K27me3, the TrxG/MLL complex catalysed the trimethylation of H3K4, switching gene transcription on and maintaining activation.^29^

In more systematic research, evidence has suggested that Fendrr coordinates bi‐directional regulation by serving as a molecular scaffold to recruit multiple chromatin regulators to regulatory elements, thereby balancing Fendrr target gene expression in a spatiotemporal manner, during mesoderm differentiation.^27^, ^28^

##### 4.1.1.2 LncRNA interactions with chromatin‐remodelling factors

Small amounts of lncRNAs have been reported to interact with chromatin remodelers, such as the chromatin‐remodelling NURF complex, the INO80 complex and the Brg1/Brm‐associated factor (BAF).^30^, ^31^, ^32^ BRG1 is a necessary component of BAF, a crucial chromatin‐remodelling complex, and is also essential for promoting embryonic heart development and pathological hypertrophy in the adult heart. In embryos, BRG1 maintains foetal cardiac growth and differentiation by interplaying with its embryonic partners, that is, histone deacetylase (HDAC) and poly ADP‐ribose polymerase (PARP). In the adult heart, BRG1 is inactivated. Interestingly, the BRG1‐HDAC‐PARP complex is reactivated under cardiac stress in adults, to trigger a pathological Myh6 to Mhy7 shift, thereby leading to aberrant cardiac differentiation and hypertrophy in mouse cardiomyocytes.^31^ Pei *et al* identified a cluster of cardiac‐specific lncRNAs, myosin heavy‐chain‐associated RNA transcripts, collectively called Mhrt, encoded from the Myh7 locus in mice cardiac issue. Myh7 levels are decreased under pathological stress, thereby contributing to abnormal gene expression and cardiac growth whereas Mhrt overexpression reverses the hypertrophic responses. Further studies have shown that Mhrt lncRNA exerts cardioprotective effects by combining with the BRG1 helicase domain, which is vital for tethering BRG1 to genomic targets. Mhrt sequesters BRG1 from its genomic DNA targets to impede chromatin‐remodelling and reverse stress‐induced pathological shifts from Myh6 to Myh7, protecting the heart against hypertrophy and dysfunction. Due to the dual‐binding features of Mhrt and BRG1 helicase regions, Mhrt expression is also competitively inhibited by BRG1‐HDAC‐PARP chromatin remodelers in response to pathological cardiac stress. Such a Mhrt‐BRG1 feedback loop is therefore important in regulating cardiac function. Human Mhrt is also transcribed from the Mhy7 locus and performs similar functions in myopathic hearts, suggesting a conserved lncRNA mechanism.^33^

#### 4.1.2 Chromosome conformation regulation and enhancer activity

**FIGURE 2:**
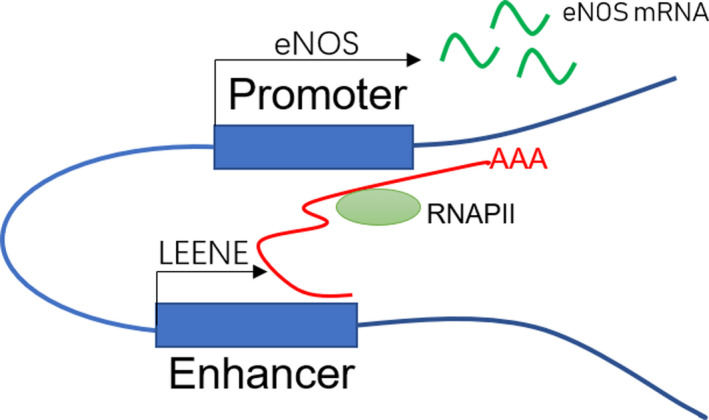
Models of enhancer activity of lncRNAs. In the chromatin looping model, lncRNA‐LEENE (red line) is transcribed from enhancer non‐coding genes. LEENE RNA transcripts recruit RNAP II to the eNOS promoter and bridge the enhancer of the non‐coding LEENE gene and the promoter of the eNOS gene. As a result, eNOS transcription levels are enhanced. lncRNA, long non‐coding RNA; eNOS, endothelial nitric oxide synthase; RNAP II, RNA polymerase II

Some lncRNAs are required for correct chromatin conformation and act through chromatin looping in enhancer‐like models.^21^ These lncRNAs appear to organize higher‐order chromatin interactions between promoters and distal enhancers, or enhancer‐like non‐coding gene loci, thereby activating transcription. These elncRNA are newly annotated lncRNAs, encoded by functional enhancers as transcriptional units.^34^ This novel class of lncRNAs orchestrates long‐range gene activation by modulating chromatin organization and structure, as recently described.^35^ These researchers identified LEENE, an enhancer‐associated lncRNA encoded in a distal enhancer region, which forms proximal associations with the endothelial nitric oxide synthase (eNOS) promoter locus. This study not only confirmed that LEENE positively regulated eNOS transcription, which is a marker of endothelial cell (EC) homeostasis and vascular function, but was also implicated in cardiovascular endothelial regulation. By combining fluorescence in situ hybridization (FISH) with chromatin conformation capture methods, chromosomal proximity associations were identified between the LEENE enhancer and the eNOS promoter in ECs. Next, they showed that LEENE RNA transcripts interacted with the LEENE‐eNOS genomic locus, to recruit RNAP II to bind the eNOS promoter, thus promoting nascent mRNA eNOS transcription. By performing multiple gain‐ and loss‐of‐LEENE functional experiments, LEENE promoted eNOS expression and eNOS‐mediated EC homeostasis, whereas LEENE deficiencies reduced eNOS mRNA levels and impaired EC function (Figure 2).

Additionally, the KCNQ1 overlapping transcript 1 (Kcnq1ot1), which is an lncRNA derived from the KCNQ1 locus, was shown to modulate Kcnq1 expression by establishing a repressive chromatin conformation.^36^ The Kcnq1 gene is essential for heart development and function, encoding voltage‐gated potassium channels in cardiac myocytes during heart development. Kcnq1 defects contribute to Long QT Syndrome (LQTS), a cardiac disorder associated with arrhythmia.^37^ Similarly, in late embryogenesis, the paternally expressed lncRNA‐Kcnq1ot1 loses its imprinted expression and converts to biallelic expression during foetal heart development. This expression occurs simultaneously with Kcnq1. In parallel with this transition, conformational chromatin changes are also detected, involving spatial proximity between the Kcnq1 promoter and distant heart‐specific enhancers.^38^ A Kcnq1ot1 deficiency leads to significant Kcnq1 expression in late embryonic heart development, an event accompanied by changes in three‐dimensional chromatin structures, and increasing chromatin flexibility and accessibility to enhancers.^36^ These findings complement chromatin regulatory mechanisms, where cardiovascular‐related lncRNAs affect transcription through mediating chromatin organization and structure.

### 4.2 LncRNAs in post‐transcriptional regulation

#### 4.2.1 LncRNAs as sources of cardiovascular miRNAs

As the two major subgroups of ncRNAs, lncRNAs and miRNAs exert significant roles in cardiovascular pathophysiology. LncRNAs serve as sources and endogenous inhibitors of miRNAs.^39^ Genomic analyses have revealed that approximately 50% of human miRNAs may be produced from the introns of coding genes, while the remainder is expressed from introns or exons of non‐coding transcripts.^10^ Initially, RNAPII transcribes miRNA genes into primary transcripts (pri‐miRNAs), which are longer hairpin‐containing RNAs.^33^ Subsequently, in the nucleus, the miRNA‐processing enzyme complex, comprised of Drosha and its cofactor Dgcr‐8, severs these pri‐miRNAs into precursors (pre‐miRNAs). These pre‐miRNAs are then transferred to the cytosol where they are processed into mature miRNAs by a second enzyme complex, Dicer and its trans‐activation response RNA‐binding protein (TRBP) complex.^40^ Thus, mature miRNAs are derived from pri‐miRNAs via the sequential processing of miRNA‐processing enzymes, Drosha and Dicer in the nucleus and cytoplasm, respectively.^41^ For example, lncRNA‐H19 has been shown to act as a primary miRNA precursor, and miR‐675 is the mature variant of the transcript.^42^ As the pri‐miRNA template for miR‐675, H19 is highly transcribed in the mouse embryo, while miR‐675 expression is restricted to the placenta. MiR‐675 inhibition was shown to be mediated by the RNA‐binding protein HuR, which binds to H19 transcripts, thereby inhibiting miR‐675 processing at the Drosha stage.^43^ Liu *et al* further confirmed that H19, as a precursor of miR‐675, was involved in the regulation of cardiomyocyte hypertrophy, by targeting calmodulin‐dependent protein kinase II d (CaMKIId). This kinase was identified as a specific target of miR‐675 and abrogated the inhibitory effects of H19 in cardiomyocyte hypertrophy. Overall, the enforced expression of H19 up‐regulated miR‐675 expression and reduced cardiomyocyte dimensions in pathological cardiac hypertrophy, whereas H19 or miR‐675 knockdown reversed these effects.^44^

#### 4.2.2 LncRNAs as negative modulators of cardiovascular miRNAs

**FIGURE 3:**
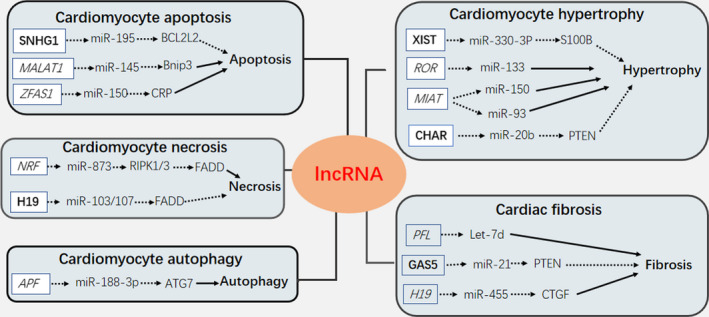
Cardiac lncRNAs, which act as miRNAs sponges, are involved in the regulation of cardiomyocyte apoptosis, necrosis, autophagy, hypertrophy and cardiac fibrosis. A solid arrow indicates promotion; a dashed arrow represents inhibition; Cardioprotective lncRNAs are in bold type; Heart‐damaging lncRNAs are in italics

LncRNAs possess mRNA‐like structures and therefore exert sponge‐like effects on multiple miRNAs to competitively sequester them from target mRNAs. Thus, lncRNAs relieve miRNA suppression on their target mRNAs, facilitating mRNA expression and function.^45^ In the study by Salmena *et al*, the competitive endogenous RNA (ceRNA) hypothesis identified a new post‐transcriptional regulatory mechanism for lncRNAs. These authors proposed that lncRNAs contained miRNA‐binding sites, also known as miRNA response elements (MREs), thus facilitating lncRNA‐binding to miRNAs.^46^ It is noteworthy that miRNAs suppress target mRNAs, by inhibiting translational activities or promoting degradation.^47^ Once miRNAs and lncRNAs are complemented, these effects are reversed, meaning that target mRNA abundance and function are up‐regulated. In a similar manner, cytoplasmic circRNAs are also sponges for miRNAs via sequence‐specific binding.^48^, ^49^ As described below, cumulative studies have shown that lncRNA and miRNA are dynamically regulated during cardiac conditions via this sponge mechanism. (Figure 3; Table 1).

**Table 1:** Overview of cardiovascular‐related lncRNA and miRNA interactions in CVDs

Interactions	LncRNA	MiRNA	Target gene(s)	Functions	Ref.
LncRNAs as sources of miRNAs	H19	miR‐675	CaMKIId	Inhibited cardiac hypertrophy progress	^44^
LncRNAs as negative regulators of miRNAs	SNHG1	miR‐195	BCL2L2	Inhibited cardiomyocyte apoptosis	^52^
	MALAT1	miR‐145	Bnip3	Increased cardiomyocyte apoptosis	54, 55
	ZFAS1	miR‐150	CPR	Promoted cardiomyocyte apoptosis	^56^
	NRF	miR‐873	RIPK1/3	Increased cardiomyocyte necrosis and I/R injury	^58^
	H19	miR‐103/107	FADD	Inhibited cardiomyocyte necrosis	^57^
	APF	miR‐188‐3P	ATG7	Promoted cardiomyocyte autophagy	59, 60
	XIST	miR‐330‐3P	S100B	Resulted in cardiac hypertrophy and cardiac dysfunction	^61^
	ROR	miR‐133		Promoted cardiac hypertrophy	^62^
	MIAT	miR‐150		Enhanced cardiac hypertrophy	^64^
		miR‐93		Promoted cardiac hypertrophy	^65^
	CHAR	miR‐20b	PTEN	Mitigate hypertrophic response	^66^
	PFL	Let‐7d		Facilitated activation of cardiac fibroblasts	^67^
	GAS5	miR‐21	PTEN	Inhibited proliferation of cardiac fibroblasts	^68^
	H19	miR‐455	CTGF	Promoted cardiac fibrosis	^69^
LncRNAs as positive regulators of miRNAs	RP5‐833A20.1	miR‐382‐5P	NFIA	Resulted in atherosclerosis	^71^
	Ang 362	miR‐221/222		Promoted VSMC proliferation	11, 72

##### 4.2.2.1 Cardiomyocyte apoptosis

Several studies have highlighted the functional role of lncRNAs as miRNA sponges in the regulation of cardiomyocyte apoptosis.^50^, ^51^ More recently, the newly identified lncRNA, the small nucleolar RNA host gene 1 (SNHG1) was shown to inhibit apoptosis in human cardiomyocytes (HCMs). A study by Ning *et al* demonstrated that SNHG1 lncRNA acted as a sponge formiR‐195 and alleviated cell apoptosis in hydrogen peroxide (H2O2)‐treated HCMs. These authors observed that miR‐195 negatively regulated the expression of BCL‐2 like protein 2 (BCL2L2), by targeting its 3’ untranslated region (3’UTR). Moreover, SNHG1 also promoted BCL2L2 expression, which was a target of miR‐195.^52^ Another cardiac apoptosis‐related lncRNA, the metastasis‐associated lung adenocarcinoma transcript 1 (MALAT1), was increased during hypoxia or ischaemia.^53^ LncRNA‐MALAT1 interacted with miR‐145 to promote oxidative stress‐associated apoptosis by inhibiting Bnip3, an initiation factor of the mitochondrial apoptotic signalling pathway. MALAT1 silencing decreased apoptosis in cardiomyocytes by H/R treatment.^54^, ^55^ Tao *et al* recently discovered a novel lncRNA, the zinc finger anti‐sense 1 (ZFAS1), which interacted with miR‐150 to block its expression, thereby up‐regulating C‐reactive protein (CRP) at mRNA and protein levels in the myocardium infarcted zones of acute myocardium infarction (AMI) mouse models. The ZFAS1 lncRNA knockdown protected cardiomyocytes against AMI by negatively mediating the miR‐150/CRP axis, via anti‐apoptosis mechanisms.^56^

##### 4.2.2.2 Cardiomyocyte necrosis

Emerging studies have shown that lncRNAs are also involved in the regulation of cardiomyocyte necrosis, by targeting miRNAs. As a major type of cardiomyocyte death mechanism in cardiac disease, programmed necrosis appears to be mediated by the receptor‐interacting serine/threonine‐protein kinases (RIPK) 1 and 3, which are antagonized by the FADD (death domain) containing Fas‐associated protein.^57^ A recent study revealed that miR‐873 diminished cardiomyocyte necrosis in the mouse ischaemic/reperfusion (I/R) model by targeting and repressing RIPK1/RIPK3 expression.^58^ To explore further molecular mechanisms, researchers searched for upstream regulators of miR‐873 and identified a novel lncRNA, necrosis‐related factor (NRF). Using a quantitative real‐time PCR (qRT‐PCR) approach, they discovered that NRF levels were increased in the ischaemic zone upon mouse I/R injury. Other studies have suggested that NRF lncRNA directly binds to miR‐873 and represses its inhibitory effects on RIPK1/RIPK3, thus intensifying RIPK1/RIPK3‐dependent programmed necrosis in cardiomyocytes. The loss‐of‐function of NRF attenuated myocardial necrosis and infarction during cardiac I/R injury. In addition, a previous study reported that lncRNA H19 competitively bound miR‐103/107, impairing FADD expression and ameliorating necrosis.^57^ Enforced expression of H19 was shown to counteract cardiomyocyte necroptosis through modulation of the miR‐103/107‐FADD pathway.

##### 4.2.2.3 Cardiomyocyte autophagy

Recent studies have suggested that lncRNAs are also implicated in the regulation of autophagy in cardiomyocytes, via several miRNAs. For example, lncRNA‐APF (autophagy promoting factor) enhanced autophagy in I/R mouse hearts by targeting miR‐188‐3p, thereby elevating autophagy‐related protein 7 (ATG7) translation, resulting in increased myocardial ischaemic injury.^59^ As well as being an important autophagy‐promoter, ATG7 is a specific target of miR‐188‐3P.^60^ By directly binding to miR‐188‐3p, lncRNA‐APF lost its cardioprotective function on autophagy through ATG7 targeting. APF knockdown exhibited prominent reductions in myocardial infarction upon I/R‐injured mice.

##### 4.2.2.4 Cardiomyocyte hypertrophy

Several research findings have highlighted important functional links between lncRNA and miRNA in terms of modulating cardiac hypertrophy. In 2018, researchers discovered that the X‐inactive‐specific transcript (XIST), which regulates X‐chromosome inactivation (XCI) in mammals, was up‐regulated in mice with hypertrophic myocardium. XIST lncRNA was shown to serve as a ‘sponge’ to miR‐330‐3p, which targeted and inhibited S100B mRNA expression. XIST elevated S100B mRNA expression levels by harbouring complementary binding sites of miR‐330‐3p. This study demonstrated that XIST overexpression alleviated hypertrophic responses in mice hearts subjected to transverse aortic constriction (TAC) and PE‐treated cardiomyocytes.^61^ Another study reported that lncRNA‐ROR (regulator of reprogramming) promoted cardiac hypertrophy via miR‐133 interactions, the expression of which was negatively correlated with lncRNA‐ROR in cardiomyocytes in response to PE treatment.^62^ Functional studies have also shown that miR‐133 plays key roles in reducing cardiac myocyte dimensions, as well as other hypertrophic hallmarks.^63^ Notably, lncRNA‐ROR down‐regulation rescued cardiomyocytes from PE‐induced hypertrophy. Emerging studies have also demonstrated the additional regulatory effects of lncRNA myocardial infarction‐associated transcript (MIAT) on cardiac hypertrophy. Zhu and colleagues suggested that lncRNA‐MIAT enhanced the pathological development of cardiomyocytes, contributing to cardiac hypertrophy development by sponging miR‐150.^64^ More recently, Li *et al*, reported that MIAT facilitated cardiac hypertrophy by sponging miR‐93 and subsequently reducing expression levels of the toll‐like receptor 4 (TLR4), a specific downstream target of miR‐93.^65^ Consistently, these studies have indicated that lncRNA‐MIAT down‐regulation attenuates cardiac hypertrophic responses, when subjected to angiotensin II (Ang II) treatment. The latest study characterized cardiac hypertrophy‐associated regulator (CHAR) as an anti‐hypertrophic lncRNA and showed it was down‐regulated in both in vivo mouse model of TAC and in vitro cellular model of cardiomyocyte hypertrophy, induced by Ang II. Moreover, miR‐20b was reported to induce cardiac hypertrophy by directly suppressing PTEN expression and indirectly aggravating AKT activity, and the level of miR‐20b could be down‐regulated by CHAR overexpression. This study showed that lncRNA CHAR absorbed miR‐20b via sponge‐like actions and mediated the miR‐20b/PTEN/AKT signalling pathway, thus mitigating hypertrophic responses.^66^

##### 4.2.2.5 Cardiac fibrosis

Several studies have reported functional lncRNA‐miRNA interactions during cardiac fibrotic processes. The pro‐fibrotic lncRNA PFL was competitively sponged to miRNA let‐7d, to exert a cardioprotective role in cardiac fibroblasts. PFL knockdown inhibited cardiac fibroblast proliferation and ameliorated cardiac functions in MI mouse models.^67^ Another anti‐fibrotic lncRNA growth arrest‐specific 5 (GAS5) up‐regulated GAS5 by targeting miR‐21 via the GAS5/ miR‐21/PTEN pathway, antagonizing cardiac fibroblast proliferation.^68^ Furthermore, lncRNA H19 was reported to play a pro‐fibrotic role in the abnormal proliferation of cardiac fibroblasts, by sponging miR‐455 and increasing connective tissue growth factor (CTGF) expression.^69^

These results unveil new mechanistic insights for lncRNAs and miRNAs, providing a novel theoretical basis for the treatment of cardiovascular disease. However, the hypothesis that lncRNAs are endogenous sponges for miRNAs is controversial. Any variation in the expression of an individual miRNA target could constitute a tiny fraction of overall target abundance. Hence, any physiological changes in the expression of individual lncRNAs may be insufficient to suppress miRNA activity. When lncRNAs are overexpressed, or cells are transfected with oligonucleotide inhibitors to dissect ceRNA interactions, these artificial procedures often go beyond the scope of physiological condition and may in fact overestimate the potential activity of a ceRNA.^70^ Moreover, further investigation is required to verify whether these functional lncRNAs are conserved in the human genome, and whether similar lncRNA mechanisms are at work in the human cardiovascular system.

#### 4.2.3 LncRNAs as positive regulators of cardiovascular miRNAs

Unlike the established negative regulation of lncRNA on miRNAs, lncRNAs also exert positive functions on miRNAs. This relationship operates during atherosclerosis and other CVDs. The lncRNA, RP5‐833A20.1, was found to be involved in the regulation of atherosclerosis development and was associated with elevated miR‐382‐5p levels, and the inhibition of nuclear factor IA (NFIA) expression. It is generally accepted that NFIA regulates cholesterol homeostasis and reverses atherosclerosis progression in CVD. Furthermore, NFIA is a direct target of miR‐382‐5p, and RP5‐833A20.1 negatively modulates NFIA expression by facilitating miR‐382‐5p expression, to significantly aggravate lipid accumulation and THP‐1 macrophage inflammation. These detrimental effects are ameliorated by lentiviral‐mediated NFIA overexpression or supplementation with miR‐382‐5p inhibitors.^71^ Regardless of these interventions, any positive correlations between RP5‐833A20.1 and miR‐382‐5p need to be experimentally clarified.

In addition, Amy *et al* identified lncRNA Ang 362, which is an Ang II‐up‐regulated lncRNA co‐transcribed with miR‐221/222 and caused vascular smooth muscle cell (VSMCs) proliferation and Ang II‐triggered endothelial dysfunction.^11^, ^72^ Using simulating proximity measurements, lncRNA Ang 362 was located proximally to miR‐221/222, indicating that lncRNAs were co‐transcribed with neighbouring genes with enhancer‐like functions.^73^ Both lncRNA Ang 362 and miRNA‐221/222 molecules were up‐regulated simultaneously, in a time‐dependent manner in response to Ang II, thereby aggravating Ang II‐induced VSMC proliferation and EC dysfunction, contributing to atherosclerotic progression. Strikingly, lncRNA Ang 362 knockdown down‐regulated miR‐221/222 expression, and attenuated VSMC proliferation, suggesting that Ang 362 manipulation could provide a novel approach for tackling atherosclerosis.

#### 4.2.4 Post‐transcriptional control of cardiovascular‐related mRNAs

Besides the well‐established ceRNAs mechanisms of action, recent studies have also reported that lncRNAs modulate mRNA alternative splicing, stability, degradation and translation efficiencies. LncRNAs primarily base‐pair with target mRNAs to form double‐stranded complexes, or they interact with RBPs to interfere with mRNA splicing and translation processes.^74^ Indeed, evidence also suggests that some lncRNAs can directly target mRNA for degradation. Blum *et al* discovered a natural anti‐sense lncRNA for tyrosine kinase containing immunoglobulin and epidermal growth factor homology domain‐1 (tie‐1AS), which was formed at the Tie‐1 locus. Tie‐1 is a receptor tyrosine kinase expressed in ECs and is essential for EC growth during vascular formation. The lncRNA tie‐1AS selectively bound tie‐1 mRNA and down‐regulated tie‐1 transcript levels resulting in EC contact junction defects.^75^ Furthermore, in the absence of base‐pairing with mRNAs, lncRNAs also interact with RBPs to interfere with pre‐mRNA splicing. For example, the nuclear lncRNA MALAT1 alters pre‐mRNA splicing patterns via interactions with serine/arginine (SR) splicing factors.^76^ Interestingly, MALAT1 depletion decreased the accumulation of SR splicing factors in nuclear speckles, while SR protein expression was increased. However, more transcriptome‐wide studies are required to understand how lncRNAs affect mRNAs in cardiovascular physiological processes.

## 5 LncRNAs COULD BE AS THERAPEUTIC TARGETS OR BIOMARKERS

With more in‐depth investigation of cardiovascular‐related lncRNAs, new molecular genetic insights can be generated on CVD biology, potentially creating unique pharmacological and therapeutic target opportunities. LncRNAs appear to regulate many cellular processes, as they act in specific tissues and cell types, making them excellent candidates for therapeutic applications.^77^ However, promising approaches targeting lncRNAs are still in their infancy; major challenges to lncRNA research are their high species‐specificity and poor conservatism. Even with major lncRNA successes in animal models, these findings are not necessarily transferable to humans. Therefore, it is particularly important to transfer the therapeutic targeting of lncRNAs from animal models to human diseases.

The lncRNA field is promising in terms of its biomarkers of various diseases in body fluids. The abnormal expression patterns of lncRNAs are often correlated with specific disease types, making these molecules appropriate for clinical diagnostics and prognostics. Therefore, circulating lncRNAs are increasingly considered as new, non‐invasive, highly sensitive biomarkers for the diagnosis, prognosis and risk stratification of CVDs.^78^ The combination of traditional biomarkers with novel lncRNA biomarkers might overcome certain deficiencies in prognostic assessments, for example, a mitochondrial‐derived lncRNA, LIPCAR was investigated in patients with and without left ventricular remodelling after MI, and was used to predict future cardiac remodelling and risk of death from HF.^79^ Recently, Zhang *et al* observed that the expression of the plasma‐based lncRNA, myosin heavy‐chain‐associated RNA transcripts (MHRT) was significantly down‐regulated in HF patients.^80^ Follow‐up studies showed that plasma MHRT levels were directly proportional to the survival conditions of HF patients. In view of this work, circulating MHRT shows promising diagnostic and prognostic use for HF treatment; however, validation in larger patient populations will be required to confirm its application as a clinical biomarker in the future.

## 6 CONCLUSIONS

Increasing evidence suggests that lncRNAs are regulated during normal and pathological cardiac physiological processes and may be clinically advantageous as therapeutic targets for the reasons previously mentioned. In this review, we focused on their mechanism of action, functional roles in pathophysiological processes and their potential as biomarkers or novel therapeutic targets in the cardiovascular system. LncRNAs regulate gene expression at the transcriptional and post‐transcriptional level through interaction with nucleic acids, and with proteins in both sequence‐ and structure‐specific manners. Our review provides an understanding of their mode of action and establishes a theoretical basis for their study as biologically relevant molecules in CVD. Indeed, more molecular insights underpinning their mechanism of action will be required to translate lncRNA research findings into clinical practice.

## CONFLICTS OF INTEREST

The authors confirm that they declared no conflict of interest.

## AUTHORS' CONTRIBUTIONS

Chengmeng Zhang: wrote the manuscript; Bing Han: prepared the Table and Figures; Dongye Li: conceived the manuscript. Tongda Xu: revised the manuscript. All authors reviewed the manuscript.

## Data Availability

Research data are not shared in this article, because no new data were created or analysed in this study.
